# A Proteomic-Based Approach to Study the Mechanism of Cytotoxicity Induced by Interleukin-1α and Cycloheximide

**DOI:** 10.1007/s10337-017-3382-3

**Published:** 2017-08-30

**Authors:** Katarzyna Macur, Jolanta Grzenkowicz-Wydra, Lucyna Konieczna, Jacek Bigda, Caterina Temporini, Sara Tengattini, Tomasz Bączek

**Affiliations:** 1 Intercollegiate Faculty of Biotechnology UG and MUG, University of Gdańsk, ul. Abrahama 58, 80-307 Gdańsk, Poland; 2 Laboratory of Pomeranian Science and Technology Park, al. Zwycięstwa 96/98, 81-451 Gdynia, Poland; 3 Department of Pharmaceutical Chemistry, Medical University of Gdańsk, al. Hallera 107, 80-416 Gdańsk, Poland; 4 Cell Biology Unit, Department of Medical Biotechnology, Intercollegiate Faculty of Biotechnology UG and MUG, Medical University of Gdańsk, ul. Dębinki 1, 80-210 Gdańsk, Poland; 5 Department of Drug Sciences, University of Pavia, Via Taramelli 12, 27100 Pavia, Italy

**Keywords:** HPLC–MS/MS, Protein disulphide isomerase (PDI), Interleukin-1α (IL-1α), Cycloheximide, Cytotoxicity, HeLa proteome

## Abstract

**Abstract:**

The exposure of HeLa cells to interleukin-1 alpha (IL-1α) in the presence of cycloheximide (CHX) leads to the release of active tumor necrosis factor alpha (TNF-α), eliciting cytocidal effect on these cells. A mass spectrometry (MS)-based analysis of the qualitative proteomic profiles of the HeLa cells treated only with IL-1α, CHX or simultaneously with IL-1α and CHX, in comparison to an untreated control, enabled to distinguish protein candidates possibly involved in this process. Among them protein disulphide isomerase (PDI) seemed to be particularly interesting for further research. Therefore, we focused on quantitative changes of PDI levels in HeLa cells subjected to IL-1α and CHX. Enzyme-linked immunosorbent assay (ELISA) was employed for determination of PDI concentrations in the investigated, differently treated HeLa cells. The obtained results confirmed up-regulation of PDI only in the cells stimulated with IL-1α alone. In contrary, the PDI levels in HeLa cells exposed to both IL-1α and CHX, where apoptotic process was intensive, did not increase significantly. Finally, we discuss how different expression levels of PDI together with other proteins, which were detected in this study, may influence the induction of cytotoxic effect and modulate sensitivity to cytotoxic action of IL1.

**Graphical Abstract:**

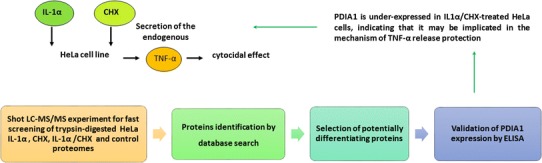

**Electronic supplementary material:**

The online version of this article (doi:10.1007/s10337-017-3382-3) contains supplementary material, which is available to authorized users.

## Introduction

Tumor necrosis factor alpha (TNF-α) and interleukin-1 alpha (IL-1α) are proinflammatory cytokines. TNF-α induces cytotoxicity in both, tumor and healthy cells. IL-1 can also trigger cytostatic or cytotoxic effect, but usually in the conditions of simultaneous treatment with metabolic inhibitors. Nevertheless, in contrast to apoptosis caused by TNF-α, which is a potent cytotoxic agent, the IL-1 activity leading to cell death is not completely understood [1, 2]. In the presence of protein synthesis inhibitor—cycloheximide (CHX), cytotoxicity was also observed in human cervical carcinoma cell line (HeLa) in response to IL-1α. There, it was dependent on the increased release of soluble and presence of transmembrane TNF-α. However, the proteins involved in this mechanism remain unknown. Interestingly, this cytocidal effect was not detected after selective IL-1α stimulation, or the effect was lower when the cells were only treated with CHX [3].

Mass spectrometry (MS)-based proteomics is a powerful approach for investigating the global changes in protein patterns characteristic for the cell, tissue or organism in certain conditions [4]. In the present research we chose to determine potential protein candidates participating in evocation of the cytocidal effect observed in HeLa cells after IL-1α and CHX treatment. We used high-performance liquid chromatography coupled with tandem mass spectrometry (HPLC–MS/MS) [5], to detect possible differences in proteomic profiles between HeLa cells in response of IL-1α/CHX, IL-1α and CHX. This allowed us to select proteins, which were detected only in one of the four analyzed proteomes. Among them, the protein disulphide isomerase (PDI) was found only in HeLa cells subjected to the IL-1α. The up-regulation of the PDI gene (P4HB) expression was shown to decrease the TNF-α gene and protein expression [6]. The PDI causes also conformational changes of the TNF-α converting enzyme—a disintegrin and metalloproteinase-17 (ADAM17). This leads to downregulation of ADAM17 enzymatic activity and prevention of pro-TNF-α processing to the secreted form [7, 8]. Therefore, PDI seemed to be particularly interesting for further examination.

In our study, we focused on quantitative changes in PDI concentrations in the investigated samples. The protein levels were measured by ELISA test in the HeLa cells exposed to both, IL-α and CHX, where the cytocidal effect was remarkable. These results were also compared to the detected PDI concentrations in an untreated control sample and in the HeLa cells stimulated alone with IL-1α, in which cytotoxicity was not observed, or treated with CHX, where it was much less pronounced [3]. This enabled comprehensive analysis of the PDI in view of its possible contribution to the TNF-α release, induced by interleukin-1α (IL-1α) and cycloheximide (CHX), which was shown to elicit the cytocidal activity on HeLa cells. Finally, the potential role in this process of other distinguished in the presented research proteins was also discussed.

## Materials and Methods

### HeLa Samples Preparation

#### HeLa Cell Culture and Induction of Cytotoxic Effect

HeLa cells (2 × 10^6^) were seeded in four 6 cm plates in 1 mL growth medium per well. They were cultured in humidified atmosphere (5% CO_2_), at 37 °C, in Dulbecco’s modified Eagle’s medium (Sigma-Aldrich, Steinheim, Germany), which was supplemented with 100 U mL^−1^ penicillin, 100 μg mL^−1^ streptomycin, 2 mM l-glutamine (Sigma-Aldrich, Steinheim, Germany) and 10% fetal calf serum (Sigma-Aldrich, St. Louis, MO, USA). They were used after 24 h at 90% confluence. Afterwards the cells were incubated:

1. alone as a control—HeLaK

or after exposure to:

2. 100 U mL^−1^ recombinant human interleukin-1 alpha (IL-1α). The IL-1α, which possessed a specific activity 3.3 × 10^7^ U mg^−1^ protein and purity >98%, was purchased at eBioscience (Vienna, Austria)—HeLaIL-1,
3. 25 μg mL^−1^ cycloheximide (CHX). The CHX was obtained from Sigma-Aldrich (St. Louis, MO, USA)—HeLaCHX,
4. both 100 U mL^−1^ IL-1α and 25 μg mL^−1^ CHX—HeLaIL-1/CHX.

After 14 h stimulation the supernatants (media) were discarded. Furthermore, the harvested cells were precipitated and the samples were lysed.

The viability of HeLa cells: after exposure to IL-1α, CHX, IL-1α/CHX and untreated HeLa control, was measured by neutral red uptake method [9] using In Vitro Toxicology Assay Kit Neutral Red Based (Sigma-Aldrich, St. Louis, MO, USA) on Multiskan FC Microplate Photometer (Thermo Scientific, Rockford, IL, USA), according to the manufacturer’s instructions.

The details of the HeLa cell culture and cytotoxicity assay are given elsewhere [3].

#### HeLa Cell Lysates Preparation

The plates with HeLa cells were placed on ice. The cells were washed 1× in PBS (prepared from Phosphate Buffered Saline Dulbecco A tablets purchased at Oxoid, Basingstoke, UK) and carefully harvested from the plate with rubber policeman in 1 mL of PBS. Next, they were placed in Eppendorf test tubes, centrifuged (1000×*g*) and supernatants discarded. The cell precipitates were lysed in 110 µL of 20 mM Tris–HCl lysis buffer (pH 7.5), which contained 150 mM NaCl, 1 mM EDTA and 1% Triton X-100 (all substances were purchased from Sigma-Aldrich, St. Louis, MO, USA). The lysates were frozen in −80 °C and sonicated after de-freezing to obtain total lysis. Then the samples were centrifuged (14,000×*g*, at 4 °C, for 20 min), supernatants collected to new Eppendorf test tubes, and stored in −80 °C prior to their analysis. The obtained protein concentrations were measured using commercially available Qubit Protein Assay Kit on Qubit 2.0 Fluorometer (Invitrogen, Calsbad, CA, USA), according to the protocol provided by the producer.

#### Protein Digests Preparation

40 µL of HeLaK, HeLaIL-1α, HeLaCHX and HeLaIL-1α/CHX lysates (0.2 mg mL^−1^) were submitted to the protein digestion. At first, 20 µL of 100 mM DTT (dithiotreitol), freshly prepared in 100 mM NH_4_HCO_3_ buffer (pH 8.5), was added to each HeLa lysate sample. Afterwards, the samples were incubated in 60 °C for 30 min to allow reduction of disulphide bridges. After cooling down to 20 °C, trypsin (100 μg mL^−1^; enzyme:substrate ratio 1:50) was added to each sample. The digestion process was performed overnight (12 h) at 37 °C. The proteolysis was then quenched by acidification with formic acid (FA). The obtained tryptic digests concentrations were 0.1 μg µL^−1^. They were then analyzed by HPLC–MS/MS on the same day.

Water used in the experiments was obtained by passing through the Direct-Q water purification system (Millipore, Bedford, MA, USA). The DTT, NH_4_HCO_3_ and FA used in these experiments were purchased at Sigma-Aldrich (Steinheim, Germany).

### HPLC–MS/MS Analysis Conditions

The HPLC–MS/MS experiments were performed on the Finnigan LTQ (Thermo Finnigan, San Jose, CA, USA) instrument. The HPLC was equipped with a thermostated column oven and Surveyor autosampler controlled at 10 °C, a quaternary gradient Surveyor MS pump and a diode array detection (DAD) system. It was combined on-line with the LTQ linear ion trap MS system with electrospray (ESI) ion source controlled by Xcalibur software 1.4 (Thermo Finnigan, San Jose, CA, USA).

The HPLC separation was performed on C-18 analytical column: XTerra MS C18 3.5 μm (2.1 × 100 mm) produced by Waters (Milford, MA, USA). Peptides elution was achieved using 120-min linear gradient from 0% B to 60% of solvent B at 200 μL min^−1^ flow rate. The mobile phases were: solvent A—0.1% FA in water, and solvent B—0.1% FA in acetonitrile, mixed on-line. All HPLC reagents were MS-grade and obtained from Sigma-Aldrich (Steinheim, Germany). The injection volume was 10 μL.

The mass spectra were generated in positive ion mode, in the 500–2000 *m/z* range, under constant instrumental conditions: source voltage 4.6 kV, capillary voltage 41 V, sheath gas flow rate 40 (arbitrary units), auxiliary gas flow 10 (arbitrary units), sweep gas flow 1 (arbitrary units), capillary temperature 220 °C, and tube lens voltage −105 V. MS/MS spectra were obtained by collision-induced dissociation in the linear ion trap, with an isolation width of 3 Da (*m/z*); the activation amplitude was 35% of ejection RF amplitude (this corresponds to 1.58 V).

*m/z* values, measured for the most intense peaks in acquired MS/MS spectra, were automatically searched against the protein database using the Sequest algorithm, incorporated into BioWorks 3.1 (Thermo Finnigan, San Jose, CA, USA). The human ‘.fasta’ format was downloaded from UniProtKB [10]. Trypsin was defined as the cleavage enzyme and up to three missed cleavages were allowed. As a result of this search we obtained peptides scoring parameters—the difference between normalized cross-correlation functions for the first and second ranked results (Δ*C*_n_) and cross-correlation score between the observed peptide fragment mass spectrum and the theoretically predicted one (*X*_corr_). To correctly identify peptides, we applied the levels of these filtering parameters evaluated by Washburn and co-workers [11] Accepted *X*_corr_ values for +1 charged fully tryptic peptides were higher than 1.9, over 2.2 or 3.3 for fully and partially tryptic +2 and +3 charged peptides. The Δ*C*_n_ values were above 0.08 for all analyzed spectra. Basing on the results obtained for each HeLa sample type, the proteins, detected only in one of four of them, were distinguished (Table 1, Online Resources ESM_1).

**Table 1:** Differentiating proteins from the investigated HeLa proteomes, identified in the HPLC–MS/MS analysis

HeLa sample	Protein name	Gene name	SwissProt Acc. #
HeLaK	26S protease regulatory subunit 6A	PSMC3	P17980
	60S ribosomal protein L7	RPL7	P18124
	ATPase inhibitor, mitochondrial	ATPIF1	Q9UII2
	CD2-associated protein	CD2AP	Q9Y5K6
	Collagen alpha-1(III) chain	COL3A1	P02461
	Filamin-B	FLNB	O75369
	Fructose-bisphosphate aldolase A	ALDOA	P04075
	High mobility group protein B1	HMGB1	P09429
	Histone H1.3	HIST1H1D	P16402
	Histone H2B type 1-C	HIST1H2BC	P62807
	Kinesin family member 13A	KIF13A	Q5JV47
	Nesprin-2 beta 2	SYNE2	Q86YP9
	Protein SET	SET	Q01105
	Serum albumin	ALB	P02768
	Taste receptor type 2 member 60	TAS2R60	P59551
HeLaCHX	40S ribosomal protein S20	RPS20	P60866
	60S ribosomal protein L21	RPL21	P46778
	60S Ribosomal protein L5	RPL5	P46777
	A disintegrin and metalloproteinase domain-containing protein 2	ADAM2	Q99965
	Aldo–keto reductase family 1 member C3	AKR1C3	P42330
	Collagen alpha-2(XI) chain precursor	COL11A2	P13942
	Endoplasmin	HSP90B1	P14625
	Immunoglobulin superfamily member 10	IGSF10	Q6WRI0
	Metalloproteinase TIKI1 precursor	TRABD2A	Q86V40
	Moesin	MSN	P26038
	Neuroepithelial cell-transforming gene 1 protein	NET1	Q7Z628
	Protein QN1 homolog	QN1	Q5TB80
	Protein sprouty homolog 2	SPRY2	O43597
	RNA-binding protein MEX3B	MEX3B	Q6ZN04
	Small glutamine-rich tetratricopeptide repeat-containing protein alpha	SGTA	O43765
	Triosephosphate isomerase	TPI1	P60174
	V3-3 protein	IGLV7-46	Q5NV83
HeLaIL-1	60 kDa heat shock protein, mitochondrial	HSPD1	P10809
	60S ribosomal protein L12	RPL12	P30050
	Argininosuccinate synthetase 1	ASS1	P00966
	ATP-citrate synthase	ACLY	P53396
	Calnexin	CANX	P27824
	Chaperonin containing TCP1, subunit 3 (Gamma)	CCT3	P49368
	Complement component 1 Q subcomponent-binding protein, mitochondrial	C1QBP	Q07021
	DNA helicase B	HELB	Q8NG08
	Extracellular sulfatase Sulf-2	SULF2	Q8IWU5
	Glutathione S-transferase P	GSTP1	P09211
	Heat shock protein HSP 90-alpha	HSP90AA1	P07900
	Hepatoma-derived growth factor	HDGF	P51858
	Heterogeneous nuclear ribonucleoprotein U	HNRNPU	Q00839
	Hydroxyacyl-coenzyme A dehydrogenase, mitochondrial	HADH	Q16836
	Keratin, type I cytoskeletal 16	KRT16	P02533
	Keratin, type II cytoskeletal 7	KRT7	P08729
	Kynureninase	KYNU	Q16719
	La-related protein 1	LARP1	Q6PKG0
	Leucine-rich repeats and immunoglobulin-like domains protein 3	LRIG3	Q6UXM1
	Mutant desmin	DES	Q45VM7
	Netrin receptor DCC	DCC	P43146
	Nucleosome assembly protein 1-like 1	NAP1L1	P55209
	Olfactory receptor 9G4	OR9G4	Q8NGQ1
	Peroxiredoxin-2	PRDX2	P32119
	Phosphoglycerate kinase	PGK1	P00558
	Poly(A) binding protein 1	PABPC1	P11940
	Poly(rC) binding protein 2	PCBP2	Q15366
	Potassium voltage-gated channel subfamily B member 1	KCNB1	Q14721
	Protein disulphide-isomerase	P4HB	P07237
	RNA-binding protein 10 isoform 2	RBM10	P98175-2
	TATA-binding protein-associated factor 172	BTAF1	O14981
	Transcriptional activator Myb	MYB	P10242
	Transient receptor potential cation channel, subfamily C, member 4	TRPC4	Q9UBN4
	Uncharacterized protein C1orf168	C1orf168	Q5VWT5
	Vinculin	VCL	P18206
	X-ray repair cross-complementing protein 6	XRCC6	P12956
HeLaIL-1/CHX	40S ribosomal protein S7	RPS7	P62081
	A disintegrin and metalloproteinase domain-containing protein 21	ADAM21	Q9UKJ8
	Chaperonin containing TCP1, subunit 2 (Beta), isoform CRA_c	CCT2	P78371
	Chaperonin containing TCP1, subunit 6A isoform a variant	CCT6A	P40227
	Homeodomain-interacting protein kinase 3	HIPK3	Q9H422
	L-lactate dehydrogenase A chain	LDHA	P00338
	LRP1 protein	LRP1	Q7Z7K9
	MHC lymphocyte antigen	HLA-G	Q9TP13
	Non-histone chromosomal protein HMG-17	HMGN2	P05204
	Non-POU domain-containing octamer-binding protein	NONO	Q15233
	Presenilin-1	PSEN1	P49768
	Protein FAM9C	FAM9C	Q8IZT9
	Protocadherin-17	PCDH17	O14917
	Tubulin beta-4B chain	TUBB4B	P68371

The details about the proteins identification can be found in the Online Resource (ESM_1)

### Functional Bioinformatic Analysis of the HeLa Differentiating Proteins

**Fig. 1:**
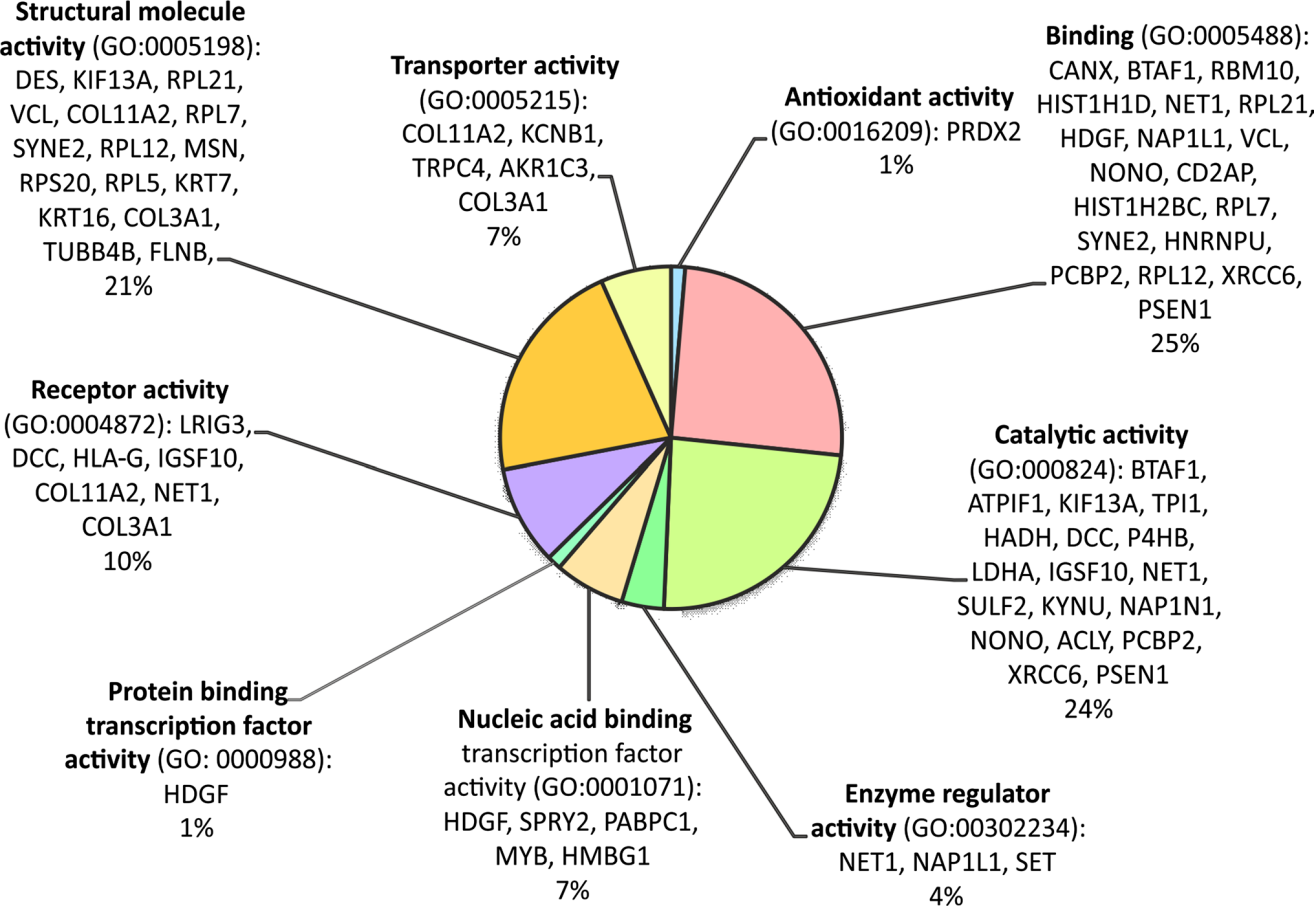
Classification of the proteins, which differentiated the investigated HeLa proteomes, according to their gene ontology (GO) molecular function categories

**Fig. 2:**
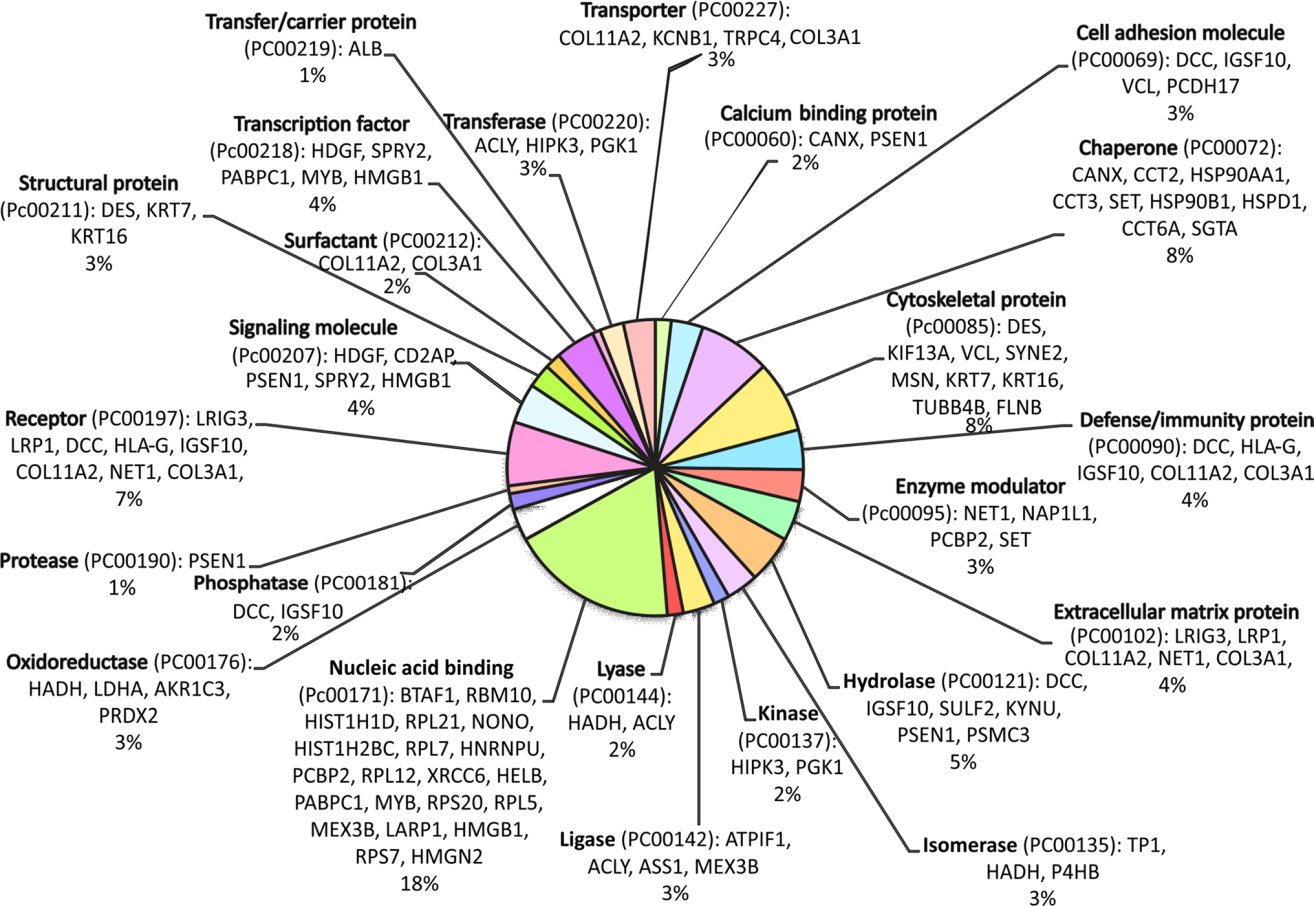
Classification of the proteins, which differentiated the investigated HeLa proteomes, according to their PANTHER protein class categories

The bioinformatic analysis of the HeLa differentiating proteins was performed with the use of PANTHER Classification System (*P*rotein *AN*alysis *TH*rough *E*volutionary *R*elationships). The gene names of these proteins were uploaded to the PANTHER Gene List Analysis tool, freely available at [12]. The proteins were classified and organized according to their gene ontology (GO) molecular function (Fig. 1) and PANTHER protein class (Fig. 2) categories. The results were depicted in pie charts. The assigned to each category proteins were also presented on sides of the pie charts.

### PDI Concentration Measurement

The verification of protein disulphide isomerase (PDI) levels in the investigated HeLa cell cultures was performed using Enzyme-linked Immunosorbent Assay (ELISA) Kit for Human Protein Disulphide Isomerase from Uscn Life Science Inc. (Wuhan, Hubei, PRC), according to the producer’s instructions. The colorimetric measurements were performed on Multiskan FC Microplate Photometer (Thermo Scientific, Rockford, IL, USA) at the wavelength of 450 nm. The assay detection range was from 0.312 to 20 ng mL^−1^. The PDI concentrations were determined then by comparing the samples O.D. to the standard curve and normalized to total protein concentration for each sample. The data were calculated as nanograms of PDI per milligram of total protein.

The obtained PDI concentrations for each type of differently stimulated HeLa cells, namely HeLaIL-1, HeLaCHX and HeLaIL-1/CHX, were then compared to the PDI concentration in the control sample (HeLaK). Statistical significance of the differences in PDI concentrations between these samples was verified using *t* Student test on Statistica 10.0 software (Statsoft, Tulsa, OK, USA). The differences were considered as statistically significant, when obtained for them *p* values were below 0.05.

## Results and Discussion

### Induction of the Cytocidal Effect on HeLa Cells

The HeLa cell cultures were stimulated with IL-1α (HeLaIL-1), CHX (HeLaCHX) or both IL-1α and CHX (HeLaIL-1/CHX). One was left as a control (HeLaK). The experiments were done according with Doszczak et al. [3]. Similarly, to their findings, the cytotoxic effect on HeLa cells in our study was observed after 14-h simultaneous IL-1α and CHX treatment.

### MS-Based Proteome Profiling

The shotgun proteomic approach was employed to investigate changes on the molecular level, in response to IL-1α and CHX that lead to TNF-α release and HeLa cells apoptosis. Tryptic digests of HeLa cell lysates were submitted to the HPLC–MS/MS analysis. The acquired MS/MS spectra were searched against protein sequence database to identify proteins in each of the samples (Online Resources ESM_1a–d). As a result of this preliminary screening, 85 peptides typical for 82 proteins (Online Resources ESM_2), which potentially diversified the examined proteomes, were distinguished. The comparison of the obtained proteomic profiles enabled to select proteins, which were found only in the control HeLa cells—HeLaK (15), in HeLa cells treated with IL-1α—HeLaIL-1 (36) or with CHX—HeLaCHX (17) and in the ones subjected to simultaneous IL-1α and CHX stimulation—HeLaIL-1/CHX (14) (Table 1, Online Resources ESM_1a–d and ESM_2). These proteins may be candidates for further detailed examination in view of their role in the mechanism of endogenous TNF-α secretion upon the co-influence of IL-1α and CHX. In the group of the proteins that differentiated the analyzed proteomes only protein disulphide isomerase (PDIA1_HUMAN) and complement component 1 Q subcomponent-binding protein mitochondrial (C1QBP_HUMAN) were identified basing on two characteristic peptides. For other differentiating proteins, there were only single characteristic peptides detected on the applied level of confidence. Those single peptide identifications should be considered with caution and require additional validation, e.g. with ELISA test, western blot or targeted for them quantitative LC–MS/MS analysis to unambiguously determine their expression levels in the samples. The distinguished proteins represented various groups and were characterized by different functions. To get comprehensive understanding of their complex nature and explore their relationships, a bioinformatic tool—the PANTHER Classification System was employed [13]. The classification results were presented in pie charts, which represent: the molecular functions of these proteins (GO Molecular Function, Fig. 1) and the protein classes (PANTHER Protein Class, Fig. 2).

### Influence of Protein Disulphide Isomerase (PDI) on TNF-α Mediated Cell Death

Among the proteins selectively identified in one of four investigated HeLa proteomes, a protein disulphide isomerase (PDI, SwissProt ID: PDIA1_HUMAN, SwissProt accession number: P07237, EC = 5.3.4.1) seems to be particularly interesting for future research. This multifunctional protein, localized in endoplasmic reticulum (ER), plays important biological roles by catalyzing formation, breakage and reorganization of disulphide bonds, and participating in the cell redox homeostasis [14]. The research of Zhou and co-workers [6], in which they induced sepsis in rats by application of lipopolysaccharide, revealed that PDI (P4HB) gene expression was lower, whereas the one encoding the TNF-α increased. In turn, TNF-α, together with other proinflammatory cytokines (e.g. IL-β or IL-6) is crucial in the initiation and progression of sepsis. It was also noticed that addition of bacitracin—a specific PDI inhibitor, to the RAW 264.7 mouse macrophages cell line can cause an increased expression of the gene encoding TNF-α in a dose-dependent manner. The conformational changes of the TNF-α converting enzyme—a disintegrin and metalloproteinase-17 (ADAM17) caused by PDI lead also to downregulation of ADAM17 enzymatic activity and prevent pro-TNF-α processing to the secreted form [7, 8]. Moreover, inhibition of PDI was shown to prevent unfolded protein response (UPR), induced by endoplasmic reticulum stress. The UPR is an adaptive intracellular mechanism of response to protein misfolding implicated in various pathophysiological states, including cancer or neurodegenerative diseases [15]. Its inhibition led to apoptosis of A375 human melanoma cell line. Therefore, PDI was indicated as a potential target for drugs, which enhance the anticancer therapy effectiveness [16]. Interestingly, it was also evidenced that PDI contributes to the increase of viability of the leukemia cell lines (HL 60 and NB4 AML) treated with daunorubicin—a cytostatic anthracycline used in cancers therapies. Instead, their additional treatment with cycloheximide caused cell death [17]. PDI belongs to the proteins that occur in rather high amounts in cells. Therefore, lack of its detection in our HPLC–MS/MS experiment in other analyzed cell lines, especially in the control HeLaK, might be an effect of the presence of other, more abundant proteins, which had, therefore, more chances to reach the MS detector during the analysis. In turn, the fact of its identification in HeLa cells stimulated with IL-1α (Table 1), in which cytotoxic effect was not observed, may indicate that cellular stress induced by this cytokine causes UPR activation and increases PDI synthesis. However, in the condition of supplemental protein synthesis inhibition by CHX (HeLaIL-1/CHX), in which an apoptotic process was intensive, this repairing mechanism could be disturbed, hence more probable lack of PDI detection.

### Protein Disulphide Isomerase is Up-regulated in HeLa Cells Stimulated Only with IL-1α

To verify quantitative relationships between PDI levels in the control HeLa cells (HeLaK) and the cells upon stimuli with IL-1α (HeLaIL-1), cycloheximide (HeLaCHX) or both of these agents (HeLaIL-1/CHX), the ELISA test was performed. The PDI concentrations in the cellular supernatants, obtained after cell lysis, were determined using a human specific ELISA and titrations of human PDI as the standard to calibrate the system. As expected, PDI could be detected in all investigated cells, but in varied levels. The average PDI concentrations were: 42.67 (±27.22) ng mg^−1^ of total protein for control HeLaK, 79.20 (±7.50) ng mg^−1^ of total protein for HeLaIL-1, 55.31 (±21.50) ng mg^−1^ of total protein for HeLaCHX and 50.55 (±18.52) ng mg^−1^ of total protein for HeLaIL-1α/CHX. Increased levels of PDI protein were present in the detergent soluble fractions of HeLa cells treated with IL-1α. The statistical analysis of the results proved that the PDI level in HeLaIL-1 was significantly higher than the level of this protein in the non-stimulated cells (*p* = 0.046 and *t* = 2.632). Furthermore, it was also higher than the PDI concentration in the HeLaIL-1/CHX cells (*p* = 0.035 and *t* = 2.869). In contrast, comparing to the control, the PDI levels were not significantly enhanced in the detergent soluble fractions of HeLa cells treated alone with CHX (*p* = 0.546 and *t* = 0.662) or simultaneously with IL-1α and CHX (*p* = 0.699 and *t* = 0.415). On the level of significance *p* = 0.05, also the PDI levels between HeLaCHX and HeLa IL-1 (*p* = 0.083 and *t* = 2.078) did not varied significantly. These data suggest that the up-regulation of PDI in the HeLa cells exposed to IL-1α may contribute to the protection against apoptosis. Next, in case of the IL-1α and CHX-treated cells, where the increase of the PDI concentration was not significant, the stress caused the release of cytotoxic TNF-α.

### Characterization of PDI in Relation with Other Proteins Differentiating the Investigated HeLa Proteomes: Potential Implication of Unfolded Protein Response and Caspase Pathway in IL-1α and CHX-induced Cytotoxicity

PDI, together with other cooperating proteins, may prevent cell death mediated by TNF-α. One of them may be calnexin (CALX) detected in the presented experiment in HeLaIL-1 cells (Table 1, Online Resources ESM_1). CALX retains unfolded glycoprotein intermediates through oligosaccharide moieties, until they fold correctly, or are being degraded [18]. Except PDI, nascent proteins synthesized in ER interact with other folding enzymes. These ER chaperones are also capable of compensating the lack of some of their representatives by up-regulating of the others [19]. The hypothesis of the ER stress pathway contribution to the mechanism of IL-1α and CHX-triggered cytocidal effect on HeLa cells, may be supported by the endoplasmin (HSP90B1) occurrence in the HeLaCHX. It is also a member of the ER protein folding control machinery and a hallmark of UPR. The protein is also an obligate chaperone for insulin growth factor (IGF), thereby participating in the IGF-driven UPR potentiating and mediated by it anti-apoptotic signalling [20]. Therefore, HSP90B1 identification in the HeLaCHX cells (Table 1) may suggest that in these cells the ER stress response mechanism was still active. Thereby, the cytotoxic effect was visible in the cells treated with CHX, although in a lower degree than in HeLaIL-1/CHX cells.

In HeLa cells, exposed to IL-1α and CHX, two other interesting proteins were detected: presenilin 1 (PSEN1) and protocadherin 17 (PCDH17) (Table 1). PCDH17 acts as a tumor suppressor. The protein causes anti-proliferative activity through induction of both, apoptosis and autophagy. Similarly, to several other members of cadherin superfamily (e.g., cadherins CDH1 and CDH11, protocadherins PCDH8 and PCDH10), functional inactivation or expression loss of this protein has been shown in various cancer types, such as gastric and colorectal cancer [21], resulting in tumor cell invasion and metastasis. The silencing of PCDH17 expression was observed in case of oesophageal squamous cell carcinoma (ESCC) [22] as well. Conversely, in normal oesophageal tissues and other tissues with squamous epithelial cells, such as cervix or skin, PCDH17 expression was present or higher than in cancerous ones. Results of these experiments suggested that PCDH17 contributes to the arrest of ESCC cells at the G_1_–S checkpoint. The PSEN1 is also involved in the control of G_1_ to S cell cycle phase transition. The loss of this negative regulator of β-catenin signalling, and the resulting further cyclin D1 transcriptional activity, was shown to lead to the epidermal hyperplasia and skin tumors development in adult mice [23]. Therefore, detection of PCDH17 and PSEN1 in the HeLaIL-1/CHX cells may suggest their lower proliferative activity, which is more characteristic to normal, non-cancerous cells. The two other proteins found exclusively in the HeLa cells treated simultaneously with IL-1α and CHX–MHC lymphocyte antigen (HLA-G) and tubulin beta-4B chain (TUBB4B) may be considered as hallmarks of the TNF-α cytotoxic activity. This may be supported by the fact that TUBB4B is one of the most abundant β-tubulin isotype in many tumors [24]. It is also worth noting that Abd-El-Basset and co-workers in the research on microglia [25] proved that the amount of total tubulin increases after treatment with TNF-α and IL-1β. Therefore, in our case, the detection of TUBB4B in the HeLaIL-1/CHX, but not in other studied HeLa proteomes, seems to be dependent on the increased expression of this protein, as a result of the IL-1α stimulation and TNF-α activity. In turn, HLA-G is known to play a role in cancer immunoediting process. The aberrant expression of this molecule by tumor cells has been suggested to be implicated in the strategies that they use to escape from the host immunosurveillance [26]. It was also shown, however, that soluble HLA-G stimulates the release of TNF-α to the culture media of peripheral blood mononuclear cells (PMBCs) incubated with membrane HLA-G expressing cells and soluble HLA-G [27]. What is more, Chen et al. [28] proved that up-regulated soluble HLA-G (sHLA-G) expression on PMBCs in ankylosing spondylitis correlates with acute phase reactants and decreases after the TNF-α blocker therapy. On the other hand, the HLA-G molecule might be also considered as a factor participating in the IL-1α and CHX-elicited, and mediated by the release of TNF-α the HeLa cells cytotoxicity. Fons et al. proved that soluble HLA-G induces endothelial cells apoptosis, which implicates caspase pathway. In their experiments, the incubation of HUVEC cells with recombinant sHLA-G1 induced these endothelial cells apoptosis, while the addition of broad-spectrum caspase inhibitor, zVAD-fmk, blocked this effect [29]. The possible implication of caspase pathway in IL-1α-induced HeLa cells killing is consistent with the previous experiments [3]. They confirmed that cells death caused by both, IL-1α and TNF-α, can be antagonized by the above-mentioned zVAD-fmk inhibition of caspases. In conditions, in which protein misfolding can no longer be compensated, the extended UPR elicits apoptosis just by the caspase pathway [30]. This could explain, in our experiment, the presence of the endoplasmic reticulum stress response proteins, as well as the ones involved in mediated by caspases cascade programmed cell death.

## Conclusions

The applied HPLC–MS/MS qualitative proteomic approach enabled to find candidates for further investigation on the nature of the mechanism of enhanced biological cytotoxic activity of TNF-α on HeLa cells after exposure to IL-1α and CHX. Among potentially differentiating proteins, a protein disulphide isomerase expression was validated using ELISA test. Presence of the other differentiating proteins, especially in case of single peptide identifications, should be considered with caution and remains to be validated, e.g., using ELISA, western blot or targeted LC–MS/MS approach, in future experiments. The proteins identified in this study suggest that the unfolded protein response mechanism together with caspase pathway may be involved in this process. Determined by the ELISA test, up-regulation of protein disulphide isomerase (PDI) indicates that this protein may be implicated in the mechanism, which protected the HeLa cells exposed to IL-1α against TNF-α release. It was disturbed, when the HeLa cells were stimulated additionally with cycloheximide. The PDI levels in HeLaIL-1/CHX cells did not increase significantly and the TNF-α secretion led to their apoptosis. Further work could also help to elucidate more precisely the quantitative relations between PDI and other proteins cooperating in this process.

## Electronic supplementary material

Below is the link to the electronic supplementary material.

Supplementary material 1 (XLSX 6974 kb)

Supplementary material 2 (XLSX 22 kb)
